# An overview of actionable and potentially actionable *TSC1* and *TSC2* germline variants in an online Database

**DOI:** 10.1590/1678-4685-GMB-2023-0132

**Published:** 2024-02-19

**Authors:** Arthur Bandeira de Mello Garcia, Guilherme Danielski Viola, Bruno da Silveira Corrêa, Taís da Silveira Fischer, Maria Clara de Freitas Pinho, Grazielle Motta Rodrigues, Patricia Ashton-Prolla, Clévia Rosset

**Affiliations:** 1 Hospital de Clínicas de Porto Alegre, Centro de Pesquisa Experimental, Laboratório de Medicina Genômica, Porto Alegre, RS, Brazil.; 2 Universidade Federal do Rio Grande do Sul, Departamento de Genética, Programa de Pós-Graduação em Genética e Biologia Molecular, Porto Alegre, RS, Brazil.; 3 Centro Universitário CESUCA, Cachoeirinha, RS, Brazil.; 4 Universidade Federal do Rio Grande do Sul, Programa de Pós-Graduação em Ciências Médicas, Porto Alegre, RS, Brazil.; 5 Hospital de Clínicas de Porto Alegre, Serviço de Genética Médica, Porto Alegre, RS, Brazil.

**Keywords:** ClinVar database, conflicting variants, TSC1, TSC2, variants of uncertain significance

## Abstract

Tuberous Sclerosis Complex (TSC) is caused by loss of function germline variants in the *TSC1* or *TSC2* tumor suppressor genes. Genetic testing for the detection of pathogenic variants in either *TSC1* or *TSC2* was implemented as a diagnostic criterion for TSC. However, TSC molecular diagnosis can be challenging due to the absence of variant hotspots and the high number of variants described. This review aimed to perform an overview of *TSC1/2* variants submitted in the ClinVar database. Variants of uncertain significance (VUS), missense and single nucleotide variants were the most frequent in clinical significance (37-40%), molecular consequence (37%-39%) and variation type (82%-83%) categories in ClinVar in *TSC1* and *TSC2* variants, respectively. Frameshift and nonsense VUS have potential for pathogenic reclassification if further functional and segregation studies were performed. Indeed, there were few functional assays deposited in the database and literature. In addition, we did not observe hotspots for variation and many variants presented conflicting submissions regarding clinical significance. This study underscored the importance of disseminating molecular diagnostic results in a public database to render the information largely accessible and promote accurate diagnosis. We encourage the performance of functional studies evaluating the pathogenicity of *TSC1/2* variants.

## Tuberous sclerosis: Epidemiology and symptomatology

Tuberous Sclerosis Complex (TSC) is an autosomal dominant genetic disorder with multisystemic manifestations associated with pathogenic germline variants in *TSC1* (OMIM ID: 605284) or *TSC2* genes (OMIM ID: 191092) (Curatolo et al., 2008). The estimated incidence of the disease varies from 1:6,000 to 1:10,000 (Osborne et al., 1991; O’Callaghan et al., 1998; Ebrahimi-Fakhari et al., 2018; Northrup et al., 2021). Patients with TSC have a broad spectrum of clinical manifestations, including hamartoma formation in different organs, commonly in the skin, kidney, and central nervous system (Northrup *et al.*, 2021). In addition to hamartomas, central nervous system manifestations include autism, epilepsy, and cognitive impairment (Crino et al., 2006) and cutaneous manifestations include angiofibromas, hypopigmented macules, shagreen patches, and confetti lesions (Northrup *et al.*, 2013; Dimario et al., 2015). These symptoms appear in different lifetime periods. In childhood, the development of central nervous system tumors and renal tumors is not uncommon, and typical tumors are subependymal giant cell astrocytomas (SEGA) (Kotulska et al., 2014; Tsai et al., 2016) and angiomyolipomas (Warncke et al., 2017; Kingswood et al., 2019). In adolescence, angiofibromas are prevalent (Liu et al., 2015) and renal cell carcinoma is more frequently diagnosed in adulthood (Yang et al., 2014; Henske et al., 2021). Loss of heterozygosity (LOH) is usually required for TSC tumorigenesis following the Knudson theory of two events (Knudson, 1971). 

## Hamartin-tuberin Complex

The *TSC1* gene (NG_012386.1) at chromosome 9q.34.4 comprises 23 exons and 60,286 base pairs (bp). The first two exons are not transcribed. The larger gene transcript contains 8,598 bp (NM_000368.5) and a long 3’-untranslated region (4,887 bp). Alternative splicing is common and there are many alternative isoforms (Carbonara et al., 1994; Van Slegtenhorst et al., 1997; NCBI). The protein product is called hamartin, with 1,164 amino acids (NP_000359.1) and a well-characterized coiled-coil functional domain (exons 17 to 23) in the C-terminal region (Santiago Lima et al., 2014; Gai et al., 2016). The N-terminal domain contains 265 amino acids forming a potential transmembrane domain (TMD) encoded by a conserved region of the gene (exon 6) (Ali et al., 2005).

The *TSC2* gene (NG_005895.1) is localized at chromosome position 16:q13, and consists of 42 exons and 46,814 bp. Like *TSC1*, it exhibits alternative splicing, producing many different isoforms, of which NM_000548.5 is the largest mRNA reference sequence (5,632 bp). It also presents several shorter transcripts with alterations in the 5’UTR (NM_001318829.2) and 3’UTR (NM_001114382.3). Exons 2-42 of the largest isoform encode the tuberin protein of 1,807 amino acids (NP_000539.2). The C-terminal region is the most conserved (Maheshwar et al., 1997), but variations in the C-terminal or N-terminal regions may occur due to previous alternative splicing (NM_001318832.2), representing the complexity and diversity of the *TSC2* gene transcripts (Duan et al., 2022; Liu et al., 2022; NCBI). 

Hamartin, tuberin, and Tre2-Bub2-Cdc16 (TBC) 1 domain family member 7 (TBC1D7) form the TSC1-TSC2 (hamartin-tuberin) protein complex, which negatively regulates the mechanistic target of rapamycin (*mTOR*) complex 1 (mTORC1), providing the proper control of different cell processes, such as cell growth and proliferation, protein synthesis and autophagy (Santiago Lima et al., 2014; Saxton and Sabatini, 2017; Zou et al., 2020). Activation of the mTORC1 pathway leads to a phosphorylation cascade in different proteins and positively regulates cell growth and proliferation. Through mTORC1 regulation, the hamartin-tuberin complex has a tumor suppressor function (Jozwiak, 2006). The hamartin coiled-coil domain is the binding region of hamartin with tuberin, and stabilizes the complex by interacting with the third member of the complex, TBC1D7 (Santiago Lima *et al*., 2014; Gai et al., 2016). The potential TMD is located in the N-terminal region of hamartin, which also appears to regulate complex stability and subcellular translocation (Hoogeveen-Westerveld et al., 2010), but the exact function of this domain is still unknown. The C-terminal region of tuberin contains the GTPase-activating protein (GAP) domain (encoded by exons 34-38, amino acids 1,531 to 1,758) (Yang et al., 2021) and the N-terminal region of tuberin (exons 2 to 12, amino acids 1 to 420) interacts with hamartin (Zech et al., 2016; Hansmann et al., 2020).

The GAP domain in tuberin has an important GTPase activity as it hydrolyses the GTP molecule associated with the Rheb protein. When Rheb associates with GTP, it stimulates the mTORC1 pathway, whereas when GTP is hydrolysed by the tuberin GTPase activity, the mTOR pathway is inhibited. The balance between mTORC1 activation and inhibition requires fine regulation, and thus patients with pathogenic variants in the *TSC1* or *TSC2* genes have a loss of function (LOF) of the hamartin-tuberin complex, leading to hyperactivity of the mTORC1 pathway. Hyperactivation of the mTORC1 pathway leads to deregulation of various cell functions, resulting in continuous cell proliferation, prolonged cell survival, and also inhibition of autophagy, which plays a central role in tumorigenesis and cancer metabolism (Kohrman, 2012; Deleyto-Seldas and Efeyan, 2021).

## Genotype-phenotype correlations

Genotype-phenotype correlations are not well established in TSC. Some examples that have been described in the literature include the occurrence of *TSC2* variants and earlier onset of epilepsy (Alsowat et al., 2021), and epilepsy with an intellectual deficit (Dabora et al., 2001; Sancak et al., 2005; Au et al., 2007; Farach et al., 2019). Interestingly, patients with the TSC phenotype but no identifiable germline pathogenic variant often have milder systemic or neurological manifestations (Alsowat *et al*., 2021). In general, alterations that result in decreased tuberin function are related to more severe symptoms (Dabora *et al*., 2001; Sancak *et al*., 2005; Alsowat *et al*., 2021). Furthermore, variants detected in the flanking regions of the *TSC2* gene and not in middle regions (exons 22-33) are related to a high risk of infantile spasms (Van Eeghen et al., 2013), and patients with *TSC1* alterations tend to show more symptoms of an anxiety disorder and minor autism manifestation (Muzykewicz et al., 2007). Regarding kidney manifestations, large deletions encompassing *TSC2* and the adjacent gene *PKD1* result in polycystic kidney disease (Oyazato et al., 2011; Boronat et al., 2014).

Considering specific *TSC1* and *TSC2 (TSC1/2)* variants, genotype-phenotype correlations have been described for only a few, such as *TSC2* c.3106T>C and *TSC2* c.2714G>A, which have been previously associated with seizures (O’Connor et al., 2003) and with mild disease (Jansen et al., 2006), respectively. Further studies are needed to make these correlations more robust and to identify other genotype-phenotype correlations.

## Molecular diagnosis in tuberous sclerosis complex

The clinical diagnosis of TSC has been established by the International Tuberous Sclerosis Complex Consensus Group (Northrup et al., 2013). As TSC is a condition with high phenotypic variability, and symptoms develop at different stages of life, some patients may not fulfill the clinical criteria for a definitive diagnosis at any given time (Northrup *et al*., 1993; Northrup *et al*., 2013). Therefore, genetic testing is a crucial part of the diagnosis, as the presence of a pathogenic variant in the *TSC1* and *TSC2* genes results in a definitive diagnosis.

Molecular diagnosis of TSC is mainly performed by next-generation sequencing (NGS) using *TSC1* and *TSC2* panels, and although copy number variants (CNV) analysis used to be performed mainly by multiplex ligation-dependent probe amplification (MLPA) (Rosset et al., 2017), more recent protocols include CNV in the NGS analysis (Singh et al., 2021). In addition, whole exome sequencing (WES) is becoming more accessible. Its widespread use in the diagnosis of conditions including cognitive impairment has led to the detection of *TSC1* and *TSC2* variants in patients with less obvious clinical features and no family history of the disease (Kovesdi et al., 2021).

Germline genetic panel testing or WES results in the identification of multiple variants and therefore a careful interpretation of NGS findings is crucial to identify causal variants (Northrup et al., 2013). A variant classification guideline was proposed by the American College of Medical Genetics and Genomics (ACMG) in 2015 (Richards et al., 2015) and optimized in 2017, with the publication of the Sherloc classification criteria (Nykamp et al., 2017). Both guidelines provide a set of criteria to classify germline variants as pathogenic (P), likely pathogenic (LP), benign (B), likely benign (LB), and of uncertain significance (VUS). Broad evidence categories used to assess the pathogenicity of a variant are the type of variant, frequency in general genetic population databases, segregation analysis, *in silico* prediction tools, functional assays, and general clinical data (Richards *et al*., 2015). If the available evidence is insufficient to accurately determine the pathogenicity of the variant, it remains as a VUS. Each specific guideline differs in the weight of evidence, thresholds, and methods, such as semi-quantitative scores or Bayesian frameworks (Nykamp *et al*., 2017; Tavtigian et al., 2018). Currently, there is no specific variant classification guideline for *TSC1* and *TSC2* genes.

## Challenges in molecular diagnosis in tuberous sclerosis complex

According to the literature, molecular testing for *TSC1* and *TSC2* in individuals with a clinical suspicion of TSC yields a pathogenic variant detection rate of 75-90% in different countries (Au et al., 2007; Northrup et al., 2013; Rosset et al., 2017; Reyna-Fabián et al., 2020; Rosengren et al., 2020; Alsowat et al., 2021; Meng et al., 2021). Among the 10-25% of patients with no pathogenic variant identified, there are a few possibilities to explain the presence of clinical symptoms, including the presence of functional variants in non-coding sequences, mosaicism, and the presence of VUS with potential to be reclassified as pathogenic (Tyburczy et al., 2015). The global median rate of VUS detection in mutation analysis studies of TSC patients is 5%. However, rates vary widely between countries: 8.0% in Brazil (Rosset *et al*., 2017), 6.5% in United States (Au *et al*., 2007), 9.8% in Canada (Alsowat *et al*., 2021), 1.6% in Mexico (Reyna-Fabián *et al*., 2020), 3.6% in Denmark (Rosengren *et al*., 2020) and 0.7% in China (Meng *et al*., 2021). Furthermore, alterations in regions that are not commonly covered by *TSC1* and *TSC2* NGS or CNV assays may be associated with disease, such as regulatory regions, promoters, and deep intronic sequences. Thus far, deep intronic mutations have already been identified in *TSC2* with the potential for a more thorough evaluation (Mayer et al., 2000; Nellist et al., 2015; Tyburczy *et al*., 2015).

The high processivity and decreasing cost of sequencing by NGS led to an increase in the discovery and accumulation of novel variants in a variety of genes, including *TSC1* and *TSC2*. However, the ability of many laboratories to conduct functional, segregation, populational, and *in silico* studies to evaluate the pathogenicity of these variants is still limited. Therefore, a great number of variants remain as VUS, with scarce clinical and functional information. VUS detection poses a significant challenge for molecular diagnosis and clinical management as it is not clear when a variant is actionable, often leading to misinterpretations and bringing distress to the carriers, their families, and even healthcare providers (Hoffman-Andrews, 2017). For example, in a study with patients with Hereditary Breast and Ovarian Cancer (HBOC) syndrome, 79% of patients who received a VUS report misinterpreted the result as a definitive predisposition to cancer (Vos et al., 2008).

One possible way to address the VUS problem is the constant performance of variant reanalysis, searching for novel evidence that could lead to variant reclassification, such as the variant frequency in updated population databases, current patient and family history data, and novel *in silico* predictions and functional assays. Variant reclassification has been reported in numerous genes with several examples in the literature (Ha et al., 2020; Iancu et al., 2021). Reanalysing VUS variants involves distinct challenges, such as: the overrepresentation of European patients in reference population databases often undermines evidence strength when evaluating variant frequency in sub-represented communities, such as Brazilian population (Gudmundsson et al., 2022); most of the reported *TSC1* and *TSC2* variants are considered rare, and detailed clinical information is often lacking (Karczewski et al., 2020); *in silico* prediction tools are a major source of discordance in variant classification, especially when using ACMG-AMP criteria (Amendola et al., 2016) and the lack of robust functional studies. Well-established functional assays are an excellent solution in variant classification and clinical molecular diagnosis, even if they require a lot of effort and have method-specific limitations.

Most of the *TSC1* and *TSC2* variants detected by genetic testing are deposited in public databases. The database with the highest number of submitted variants is ClinVar (https://www.ncbi.nlm.nih.gov/clinvar/). ClinVar is also the most used database for assessing the clinical interpretation of variants. However, the information related to the variants are controversial (carrier clinical information, variant details) and a high percentage of variants remain missing from public databases.

## Aims

The present review explores the *TSC1* and *TSC2* variants in ClinVar, a main online variant database, with three major aims: 1) to review the distribution of the reported variants; 2) to describe the level of evidence used to classify variants as pathogenic, and 3) to assess the reclassification potential of current VUS. Additionally, we provide a review of the scientific literature to describe the distinct strategies used for functional assays to analyze *TSC1* and *TSC2* variants. We intended to summarize the *TSC1* and *TSC2* variant spectrum in ClinVar, as well as point out the provided and missing evidence in variant classification details, to identify bottlenecks in this process and help in its improvement.

## Landscape of TSC1 and TSC2 variants in the ClinVar database

Up until January 04th, 2023, ClinVar had reported 3,690 variants in *TSC1* and 8,500 variants in *TSC2*. ClinVar assorts variants into the following categories: clinical significance, types of conflicts, molecular consequence, variation type, variation size, variant length and review status. In each category, there are specific filters to analyze variants. We evaluated actionable and possibly actionable variants, conflicting variants, variation type and molecular consequence.

To explore the spectrum of these data, we first downloaded the complete list of variants of both genes in Excel format. The complete genomic range of both genes was analyzed. After that, we selected all filters (conflicting, B, LB, VUS, LP, P) in the clinical significance category in ClinVar for each gene, downloaded the correspondent Excel format and compared it with the respective complete variant list. The comparisons were performed using Excel. We found that 312 alterations in *TSC1* and 877 in *TSC2* do not have their clinical significance recorded in the database (not provided - NP), and were excluded in further analysis. For the variants with a clinical significance (*TSC1*=3,378 and *TSC2*=7,623), we used the ClinVar filters B and LB simultaneously to obtain the number of unique variants, since a few alterations are submitted both as B and LB (Table 1). We repeated this process with P and LP variants. We also checked for the number of VUS and variants with conflicting submissions. We used the same strategies to evaluate the molecular consequences and variation types of the alterations reported for each gene. The molecular consequences are described as frameshift, missense, nonsense, splice site, and untranslated region (UTR). We carefully analyzed the variants that have multiple molecular consequences reported and gathered them accordingly. The variation types are described as indel, deletion, duplication, insertion, and single nucleotide variants (SNV). Variants with multiple submissions were grouped accordingly. 

**Table 1 -:** Total number of *TSC1* and *TSC2* variants according to ClinVar categories: clinical significance, molecular consequence and variation type.

	TSC1	TSC2
Clinical significance	Number of Variants	Number of Variants
Conflicting Interpretations	230 (6.23%)	664 (7.80%)
Pathogenic	418 (11.33%)	841 (9.90%)
Likely Pathogenic	64 (1.73%)	144 (1.70%)
Likely Pathogenic and Pathogenic	17 (0.46%)	42 (0.50%)
Uncertain Significance	1,533 (41.54%)	3,176 (37.40%)
Likely Benign	792 (21.46%)	1,906 (22.40%)
Benign	116 (3.14%)	144 (1.70%)
Likely Benign and Benign	208 (5.64%)	706 (8.30%)
Not Provided	312 (8.46%)	877 (10.30%)
Total	3,690 (100%)	8,500 (100%)
Molecular Consequence	Number of Variants	Number of Variants
Frameshift	364 (9.86%)	689 (8.11%)
Missense	1,376 (37.29%)	3,376 (39.72%)
Nonsense	176 (4.77%)	294 (3.46%)
Splice site	95 (2.57%)	322 (3.79%)
UTR	262 (7.10%)	106 (1.25%)
Frameshift+UTR	21 (0.57%)	27 (0.32%)
Frameshift+Splice	0 (0%)	2 (0.02%)
Missense+UTR	124 (3.36%)	131 (1.54%)
Missense+Splice site	1 (0.03%)	5 (0.06%)
Nonsense+UTR	10 (0.27%)	12 (0.14%)
Not Provided	1,261 (34.17%)	3,536 (41.60%)
Total	3,690 (100%)	8,500 (100%)
Variation type	Number of Variants	Number of Variants
Deletion	329 (8.92%)	732 (8.61%)
Duplication	34 (0.92%)	48 (0.56%)
Insertion	36 (0.98%)	53 (0.62%)
SNV	3,032 (82.17%)	7,090 (83.41%)
Indel	31 (0.84%)	89 (1.05%)
Dup+Ins	164 (4.44%)	371 (4.36%)
Dup+SNV	0 (0%)	1 (0.01%)
Dup+Del	0 (0%)	1 (0.01%)
Ins+SNV	0 (0%)	1 (0.01%)
Not Provided	64 (1.73%)	114 (1.34%)
Total	3,690 (100%)	8,500 (100%)

Table 1 summarizes the data obtained from the aforementioned analyses. In both genes, VUS is the most reported category. This reinforces the need for functional and/or clinical segregation studies to understand the role of these variants (Hoogeveen-Westerveld et al., 2011; Millot et al., 2012; Guidugli et al., 2014; Nix et al., 2020). Regarding molecular consequence and variation type categories, missense and SNVs are the majority, respectively. Notably, we found a large number of variants with no information. For example, of the total variants in *TSC1* and *TSC2,* 34.17% and 41.60%, respectively, do not have their molecular consequence reported. We analyzed the variants with missing clinical significance, molecular consequence and variation type in Excel. The terms used for excel filtering were not available in ClinVar as filters (e.g. synonymous, microsatellite and others). The available details about these variants are shown in Table S1. The majority of *TSC1* variants without clinical significance are deletions (32.69%), duplications (15.71%) and nonsense (14.74%) variants. These variants in *TSC2* are mostly deletions (27.14%), splice site (25.43%) and missense (16.65%) alterations. Moreover, many *TSC2* variants with no variant type information are represented by deletions (29.82%), microsatellite (28.95%) and splice site variants (22.81%). In *TSC1*, microsatellites are 96.5% of these variants with no variant type information. 

Next, we analyzed the molecular consequences and variation types according to their clinical significance. We first applied the clinical significance filter in ClinVar and divided variants in four groups: B/LB, P/LP, VUS and conflicting variants. Variants without a description of clinical significance were excluded from this analysis. A second simultaneous filter was applied, dividing the variants of each group by molecular consequence or by variation type. The results are summarized in Table S2. We analyzed the variants with clinical significance and no molecular consequence and/or variation type information using Excel. The terms used for excel filtering were not available in ClinVar as filters (e.g. synonymous, microsatellite and others). The results are described by group (B/LB, P/LP, VUS and conflicting variants) in Tables S3 and S4. 

**Figure 1 -:**
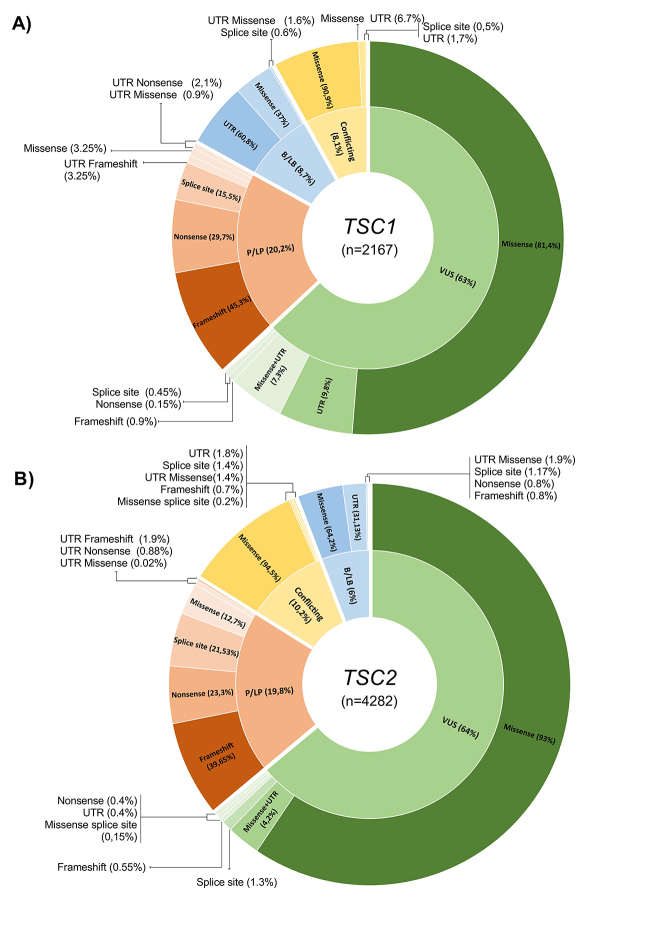
Sunburst charts representing all variants with clinical significance and molecular consequence reported in the ClinVar Database. **A)** *TSC1* variants distribution **B)** *TSC2* variants distribution. The external layer represents the variant type and the internal layer represents the clinical significance. Percentages in the internal layer are represented in relation to the total variants. Percentages in the external layer are represented in relation to clinical significance.

Finally, to visually demonstrate the spectrum of variants with clinical significance relating their effects on protein, we constructed a sunburst chart using the total variants with reported clinical significance and molecular consequence as input (*TSC1*=2167 and *TSC2*=4282) (Figure 1). The figure shows that B/LB variants have specific profiles of molecular consequence. For example, missense and UTR alterations generally categorize B/LB variants. Most 3’ and 5’ UTR alterations were described as B/LB in *TSC1* (10.30%, n=115/1116) and *TSC2* (2.90%, n=80/2756). On the other hand, missense and UTR alterations represent the minority of P/LP variants in both genes. P/LP variants also have specific profiles of molecular consequences. Nonsense, frameshift and splice site alterations represent most of these variants. Nonsense variants have strong evidence for pathogenicity, since they induce the formation of a premature stop-codon, synthesizing a truncated protein. The damage level of a nonsense variant depends on the protein lacking region. Transcripts with nonsense variants can also be a target for the nonsense-mediated decay (NMD) pathway. This pathway degrades defective mRNA, displaying a pathogenic outcome due to haploinsufficiency (Kervestin and Jacobson, 2012). Frameshift variants are also mostly pathogenic and were not detected in the B/LB spectrum, as expected. (Supplementary Table S2, Figure 1). 

Missense variants represent 72.47% (n=1,111) and 80.2% (n=2,457) of total VUS on *TSC*1 and *TSC*2, respectively. Also, missense variants present a high number of conflicting interpretations. Likewise, UTR alterations were frequently described as VUS. In general, VUS have similar molecular consequences as B/LB alterations, like missenses or UTR variations. On the other hand, a few VUS are described as having similar molecular consequences as P/LP variants: nonsense (*TSC1*=2 and *TSC2*=11), frameshift (*TSC1*=11 and *TSC2*=15), and splice sites (*TSC1*=1 and *TSC2*=36) (Supplementary Table S2, Figure 1). Therefore, VUS with these consequences need more attention, as they have the potential for reclassification as pathogenic or likely pathogenic, representing 1.3% of VUS in *TSC1* and 1.95% in *TSC2*.

## Distribution of ClinVar variants in TSC1 and TSC2 genes

**Figure 2 -:**
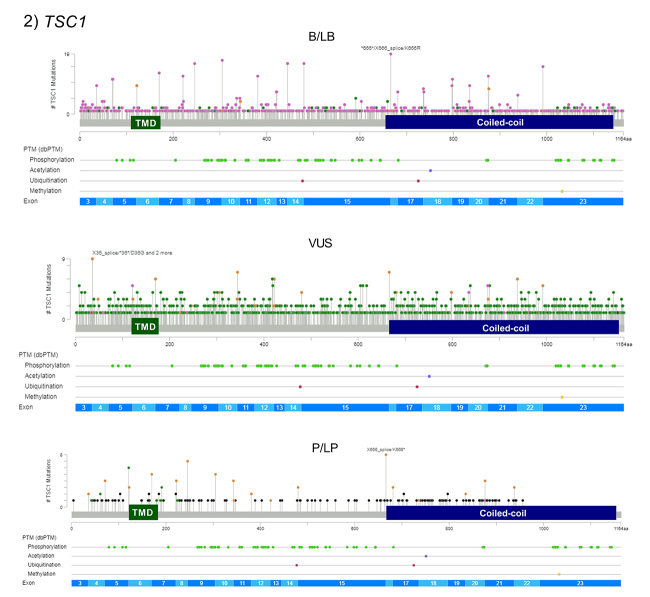
Gene distribution of *TSC1* single nucleotide variants (SNV) for benign or likely benign variants (B/LB), variants of uncertain significance (VUS), and pathogenic or likely pathogenic variants (P/LP). Intronic variants are not represented. Darker green dots represent missense variants. Yellow dots represent splice site variants. Black dots represent truncated variants (nonsense or frameshift deletions). Pink dots represent other variant types (synonymous, UTR and intronic variants). The *TSC1* gene region that codes for TMD (predicted) and coiled coil domains are represented. The post translational modifications (PTM) are shown below the graphics (lighter green represents phosphorylation sites).

**Figure 3 -:**
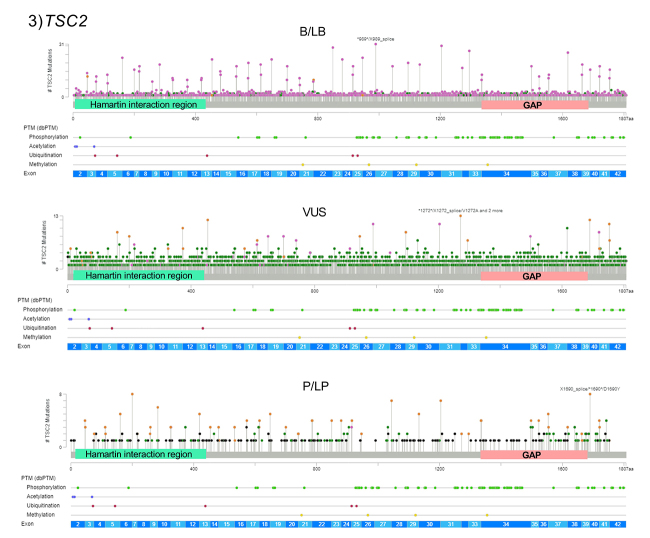
Gene distribution of *TSC2* single nucleotide variants (SNV) for benign or likely benign variants (B/LB), variants of uncertain significance (VUS), and pathogenic or likely pathogenic variants (P/LP). Intronic variants are not represented. Darker green dots represent missense variants. Yellow dots represent splice site variants. Black dots represent truncated variants (nonsense or frameshift deletions). Pink dots represent other variant types (synonymous, UTR and intronic variants). The *TSC2* gene regions that code for the Hamartin interaction region and GAP domain are demonstrated. The post translational modifications (PTM) are shown below the graphics (lighter green represents phosphorylation sites).

SNVs represent the great majority of variants in *TSC1* (n=3,032/3,690) and *TSC2* (n=7,090/8,500) (Table 1). Thus, we analyzed the distribution of SNVs along the two genes. This approach can help to identify putative hotspots for variation and clusters of variants depending on their clinical significance, such as pathogenic variants in regions that have a direct influence on protein function. Genomic localization and distribution of all SNVs (NM_000368.5 for *TSC1*; NM_000548.5 for *TSC2*) was performed using the ‘’Mutation Mapper’’ genomic tool provided by cBioPortal v5.2.0. The variant distribution is shown in Figures 2 and 3. Apparently, B/LB variants and VUS are even distributed in both genes. In *TSC2*, P/LP variants also seem to be evenly distributed. On the other hand, there are modest clusters of P/LP variants in the *TSC1* gene portion that codes for the N-terminal region and coiled-coil domain of hamartin. In additional molecular studies, most of the pathogenic variants are detected in the *TSC1* gene portion that codes for the N-terminal hamartin region, which includes the putative TSC1 TMD domain (Mozaffari et al., 2009). In other proteins, TMDs have already been associated with the transport and sorting of transmembrane proteins (Cosson et al., 2013). Menon et al. (2014) demonstrated that the TSC complex binds the lysosomal membrane in a dependent manner of the interaction with Rheb protein. However, further studies are essential to understand the functionality of this putative domain in hamartin.

In *TSC1*, exons 9-15 code the main phosphorylation sites of hamartin (Figure 2). Therefore, variants detected in these exons may alter phosphorylation in the corresponding protein and potentially alter protein function. In addition, the hamartin C-terminal region contains the coiled-coil domain involved in hamartin-tuberin binding (Santiago Lima et al., 2014). Many pathogenic SNVs are described in this region, clustered in exons 18-21. A pathogenic variant in this location could prevent hamartin-tuberin interaction, and consequently affect the GTPase activity of tuberin (Huang and Manning, 2008). Therefore, VUS in the coiled-coil domain requires greater attention, especially the frameshift, nonsense, and splice site variants.

Pathogenic variants are widespread in the most important functional regions in tuberin: the GAP domain and the sequence that interacts with hamartin. VUS detected in these regions need careful attention, as they are highly conserved domains (Maheshwar et al., 1997). Moreover, pathogenic variants that affect tuberin phosphorylation sites, such as in hamartin, occur mostly in the region between exons 33-34 (Figure 3).

**Figure 4 -:**
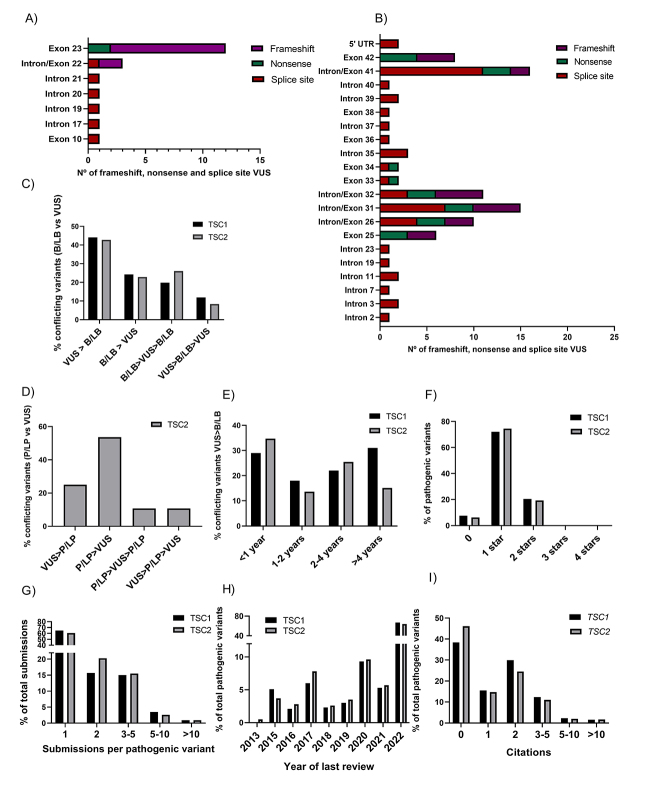
An overview of *TSC1* and *TSC2* VUS, conflicting and pathogenic variants submitted in ClinVar. **A,B)** Distribution of VUS with molecular consequence similar to pathogenic variants: frameshift, nonsense, splice site in *TSC1* and *TSC2*, respectively. **C)** *TSC1* and *TSC2* B/LB vs VUS conflicting variants were separated in four groups: VUS>B/LB, B/LB>VUS, B/LB > VUS > B/LB, VUS > B/LB > VUS; **D)** *TSC2* P/LP vs VUS conflicting variants were separated in four groups: VUS>P/LP, P/LP>VUS, P/LP > VUS > P/LP, VUS > P/LP > VUS; **E)** Representation of the time (in years) between a VUS submission and its reclassification as B/LB. **F)** Review status of *TSC1* and *TSC2* pathogenic variants; **G)** Number of submissions per *TSC1* and *TSC2* pathogenic variants in ClinVar; **H)** Year of the most recent submission of *TSC1* and *TSC2* pathogenic variants; **I)** Number of citations of *TSC1* and *TSC2* pathogenic variants.

## Variants of uncertain significance (VUS) in ClinVar: Spectrum and potential for reclassification

As previously mentioned, when the available evidence does not support a variant classification, the variant pathogenicity cannot be ascertained, and it is categorized as VUS. VUS are not informative in the prediction of disease occurrence and/or risk and are often referred to as not actionable, demanding special attention and periodic reanalysis (Chern et al., 2019). Therefore, we evaluated their review status in the ClinVar database. ClinVar reports the level of review supporting the assertion of clinical significance for the variation as review status. Stars provide a graphical representation of the variant aggregate review status. In this classification, one star is given for variants with a single submitter or conflicting interpretations (with multiple submitters); two stars when there are multiple submitters with no conflicts in interpretation; three for variants reviewed by an expert panel; and four for variants classified by specific guidelines. Variants with no stars are those not reviewed and/or with no information registered. Review statuses of *TSC1* and *TSC2* VUS and variants with conflicting interpretations (B/LB vs VUS or P/LP vs VUS) are summarized in Table 2. None of the variants described in ClinVar have three or four stars and most of the VUS have a single star. This is an important finding that highlights the lack of VUS review by ClinVar submitters and/or the lack of information registered in ClinVar.

**Table 2 -:** Review status of *TSC1* and *TSC2* VUS in ClinVar regarding number of stars.

VUS + Conflicting interpretation		
Stars^a^	*TSC1*	*TSC2*
None	2	16
★	1,123 +230**ᵇ**	2,195 + 664**ᵇ**
★ ★	408	965
★ ★ ★	0	0
★ ★ ★ ★	0	0
Total	1,763	3,840

ªNo star: no assertion provided or no assertion criteria provided; One star: criteria provided with single submitter or criteria provided with conflicting interpretations; Two stars: multiple submitters employing criteria provided no conflict interpretations; Three stars: reviewed by expert panel; Four stars: practice guideline.

ᵇVariants with multiple submitters and conflict interpretation in ClinVar.

Since VUS represent a challenge in molecular diagnostics and they are poorly reviewed in Clinvar, we analyzed the distribution of frameshift, nonsense, and splice site VUS in more detail due to their potential to be reclassified as pathogenic. The distribution of these types of VUS is shown in Figure 4A,B. In *TSC1*, a total of 95% of the analyzed VUS (19/20) are located in exons that code for the coiled-coil domain. Of these VUS, 15 are located between exons 22-23 (Figure 4A). In our previous analysis of pathogenic variant distribution, (Figure 2C), exon 22 showed few alterations, and exon 23 did not show pathogenic variants. Hence, these exons have a high VUS description and few pathogenic variants. Perhaps, this region lacks functional analyses and deserves to be explored for VUS reclassification. 

Furthermore, we investigated VUS detected in other exons that code for the hamartin C-terminal domain, i.e. exons 15 to 21. This region has a high number of pathogenic variants detected. A few VUS described in this region are predicted to affect splice sites, such as c.2209-2A>G. On the other hand, the variant c.2624_2625+3dup does not have a prediction of effect on splicing, but it is positioned at a conserved region. The variant c.2391+1G>A detected in intron 17 has no available information in ClinVar, but Varsome (version 11.8.0) classified it as likely pathogenic (Kopanos et al., 2019; VarSome). 

Regarding *TSC2*, a total of 15 VUS are located in exon 26 (E26) or exon 32 (E32), and six in intron 26 or intron 32 (Figure 4B). Six of these VUS predict nonsense alterations (E26=3; E32=3), eight frameshift (E26=3; E32=5), and three are splice site alterations (E32=3). Exon 34 is the largest *TSC2* exon and has many pathogenic variants detected (Figure 3). The variants c.4081C>T (nonsense) and c.4008_4010del (in frame deletion) are detected in this exon. The variant c.4008_4010del has three VUS submissions in ClinVar, with an inconclusive *in silico* study (Choi et al., 2012). Additionally, two splice site VUS have been identified in the GAP domain (exon 34-38) and five are located in introns 34-38. Fifty variants present only one submission and 11 have two submissions, highlighting again the lack of information about certain submitted variants.

## Variants with conflict interpretation in *TSC1* and *TSC2* in ClinVar

ACMG and Sherloc guidelines present standardized criteria for variant evaluation (Richards et al., 2015; Nykamp et al., 2017). Even though they are standardized, criteria interpretation may be subjective, which leads to variable classifications of the same variant by distinct groups. A few groups/laboratories that submit variants in ClinVar adopt standard or modified versions of ACMG-AMP and Sherloc criteria. Most of the submitters develop their own classification criteria, some of them not publicly available (Niehaus et al., 2019). The use of numerous distinct guidelines, unavailable data and methodology can undermine variant classifications and result in multiple conflicting submissions in ClinVar (Yang et al., 2017).

To analyze variants with conflicting interpretations, we filtered the ClinVar variants with the conflicts P/LP *versus* VUS and B/LB *versus* VUS (*TSC1*=230, *TSC2*=664). A total of 227 and 627 variants presents the conflict B/LB *versus* VUS in *TSC1* and *TSC2*. Three and 29 are conflicting P/LP *versus* VUS in *TSC1* and *TSC2* variants. Eight variants present multiple conflicts (B/LB vs P/LP vs VUS) in *TSC2*. Single nucleotide variation represents 222 and 646 in *TSC1* and *TSC2*, respectively. 

Further, we separated the variants with conflicting interpretations into four groups: Group 1, variants with a first submission as VUS, which were later classified as B/LB; Group 2, variants with a first submission as B/LB, which were later classified as VUS; Group 3, variants with a first submission as B/LB, second submission as VUS and a third submission as B/LB again; and Group 4, variants with a first submission as VUS, second submission as B/LB and a third submission as VUS again. We excluded only one VUS once its year of submission was not demonstrated. Variants in group 1 were possibly reclassified as B/LB in a second evaluation, depending on the employed criteria. Groups 3 and 4 represent extensive conflicting interpretations. All variants in each group are shown in Figure 4C. 

Additionally, we used the same grouping strategies to evaluate P/LP *versus* VUS conflicts in *TSC2* (n=28). In *TSC1*, only three variants present P/LP variants *versus* VUS conflicts, thus were not included in further analysis. Group 1 (n=7/664) and group 2 (n=15/664) represent the majority of *TSC2* conflicting variants (Figure 4D). Curiously, a few variants were submitted as P/LP in the first submission even without presenting the sufficient information for this classification. These variants were later submitted as VUS by other laboratories. This issue raises concern about the variant classification process by different users. The criteria for variant classification are subjective, and errors or misinterpretations might occur. Moreover, a few groups use their own criteria, many times not publicly available. Thus, ClinVar consultants should be cautious about variant classification submissions. On other hand, a few B/LB variants were submitted firstly as VUS over a lack of information and posteriorly classified as B/LB, with the rise of functional studies or other additional information to classify the variant accordingly. 

For variants with the conflict B/LB *versus* VUS, group 1 variants were filtered to examine how many years it took for VUS reclassification as B/LB. We examined the year of VUS submissions that were later submitted as B/LB (Figure 4E). It seems that *TSC2* is more often reviewed since most of its VUS (n=94/271) were reclassified as B/LB in less than a year. In *TSC1*, a significant number of variants were also reclassified in less than one year (n=29/100), but most variants took more than four years for a reclassification (n=31/100). Finally, for variants with the conflict P/LP *versus* VUS, seven *TSC2* conflicting variants were later submitted as P/LP. Five of these variants were given a second classification in less than one year and two in 2-4 years.

## Pathogenic variants in *TSC1* and *TSC2* in ClinVar

We subsequently collected information from *TSC1* and *TSC2* variants with at least one submission as pathogenic in Clinvar. Information gathered for the selected variants were review status, number of submissions, last submission date, functional evidence, and number of citations in literature. A total of 434 and 883 pathogenic variants were analyzed for *TSC1* and *TSC2*, respectively. Review status of the variants was mainly one star (*TSC1*=311; *TSC2*=654), followed by two stars (*TSC1*=88; *TSC2*=170), and an absence of variants with three or four stars (Figure 4F). Most of the variants had one submission, accounting for 64.8% of variants in *TSC1* and 61.4% in *TSC2* (Figure 4G). A high number of variants had their most recent submission in the year of 2022 (*TSC1*=290; *TSC2*=563). Except for four *TSC2* variants classified in 2013, no variants had their last submission date before 2015 (Figure 4H). There were a significant number of variants with no literature citations identified (*TSC1*=166; *TSC2*=406) (Figure 4I). Only seven variants had functional evidence available in ClinVar (*TSC1* = 2 and *TSC2* = 5).

## Strategies for functional assessment of *TSC1* and *TSC2* variants

Functional studies are important to understand the role of variants in protein function and consequently in disease. In this sense, they are crucial for VUS reclassification and/or to reinforce the classification of B/LB, P, and LP variants. To analyze the strategies used to functionally evaluate *TSC* variants, we searched the scientific literature in PubMed using the words “functional assessment”, “*TSC1*” OR “*TSC2*”, and found 431 manuscripts. Of these, only twelve studies had performed a functional assessment. 

The majority (9/12) of the functional studies found in our search used similar strategies applied by the Nellist group in 2001 and 2005 (Nellist *et al*., 2001, 2005). These strategies are the transfection of *TSC1* and/or *TSC2* defective transcripts via lipofectamine or plasmid in HEK-293 cells, followed by immunoprecipitation by immunoblotting. To confirm whether the variant in question decreases the *TSC1-TSC2* interaction and decreases the stability of the *TSC2* protein, studies evaluated the *TSC1* signal and the *TSC2* signal in immunoblotting, respectively. Furthermore, as the *TSC2-TSC1* complex has the function of inhibiting mTOR, the mTORC1 activity was analyzed by the ratio of phosphorylation of downstream proteins by immunoblot, such as T389-phosphorylation at p70 S6 kinase (*S6K*) (Hodges et al., 2001; Jansen et al., 2008; Nellist *et al*., 2008; Dunlop et al., 2011; Hoogeveen-Westerveld et al., 2011, 2012, 2013; Overwater et al., 2016; Živčić-Ćosić et al., 2017).

In addition, intronic and exonic variants may cause mRNA processing errors. For this, some studies used the cDNA analysis strategy to verify the presence of all exons or the inclusion of introns in the transcripts (5/12). This cDNA analysis consists of a RT-PCR, a method to analyze the difference of amplicon sizes of *TSC1* and *TSC2* specific primers by electrophoresis. Therefore, differences in amplicon size can show the exon absence or intron inclusion, indicating an error in mRNA processing (Kobayashi et al., 1995; Jeganathan et al., 2002; Jansen et al., 2008; Tyburczy et al., 2015; Overwater et al., 2016). Additionally, three studies (3/12) accomplished the same technique for cDNA analysis but did not perform additional functional analysis (Mayer et al., 2000; Tyburczy *et al*., 2015; Qiu et al., 2020). 

The functional assessments using cDNA could reveal alterations outside the scope of NGS and MLPA. For example, we can obtain information about RNA processing attributed to variants in cDNA analysis. Furthermore, by analyzing proteins, we can verify the presence or absence of parts of the protein, the stability and functionality of the *TSC1/TSC2* complex, and the activation of downstream proteins in the mTOR pathway. Functional assays are arduous and require a long time of laboratory activity to standardize the methodology and analysis. However, the existing *TSC1* and *TSC2* functional assays often don’t meet the requirements to be considered well-established and strong evidence (Gelman et al., 2019). Nonetheless, if a well-established *in vitro* or *in vivo* functional assay shows a variant with deleterious effect that fits in PS3 ACMG criteria, it could be supported as pathogenic evidence (Richards et al., 2015). Studies should be benchmarked with well-known pathogenic and benign variants that can fully demonstrate the dynamic range of the assay and the whole spectrum of pathogenicity in a given gene. Additionally, functional assays based on cDNA constructs lack regulatory regions and might not fully represent the endogenous situation (Brnich et al., 2019). In spite of functional assessment limitations, it is very important to perform and report these assays to better understand the role of these variants. 

## Conclusions

In this review, we explored the *TSC1* and *TSC2* ClinVar database and evaluated variant distribution, the level of evidence used to classify variants as pathogenic, and the potential of reclassification of current VUS. Up until January 4th, 2023, ClinVar had reported 3,690 variants in *TSC1* and 8,500 variants in *TSC2*. We did not observe hotspots for variation in both genes, and missense and single nucleotide variants were the majority. In addition, VUS is the most frequently reported category. This reinforces the need for functional and/or clinical segregation studies to understand the role of these variants. Our analyses revealed that in general VUS have similar molecular consequences as B/LB alterations. However, VUS described as having similar molecular consequences as P/LP variants need more attention, as they have the potential for reclassification as pathogenic or likely pathogenic. We also observed that most of the VUS lack information in their ClinVar submissions and have poor review status. This highlights the importance of complete submissions in online databases, including criteria used for variant classification, clinical, and segregation data. In addition to these issues, the use of numerous distinct guidelines, unavailable data and methodology can undermine variant classifications and result in multiple conflicting submissions in ClinVar. Indeed, we found a high number of variants with conflict interpretations: 230 in *TSC1* and 664 in *TSC2*. Of these variants, 43.4% in *TSC1* were VUS in the first submission and were later classified as B/LB (20.4% in less than two years). For *TSC2*, 40.8% were VUS in the first submission and were later classified as B/LB (19.7% in less than two years). For VUS that were later classified as P/LP, 2/230 (0.8%) were found in *TSC1* (0.4% in less than two years) and 7/664 (1.05%) in *TSC2* (0.75% in less than two years). These numbers reinforce the need for further studies to evaluate VUS with the potential for pathogenic reclassification. Functional studies are crucial for VUS reclassification and/or to reinforce the classification of B/LB, P, and LP variants. We observed a lack of these types of studies in *TSC1/2* genes. Considering all pathogenic variants, only six of the 1,211 variants submitted for both genes have functional assays to confirm pathogenicity. We found only 12 functional studies for *TSC1* and *TSC2* variants in the scientific literature. This encourages the performance of further functional studies evaluating the role of *TSC1* and *TSC2* variants in protein function. In summary, the critical analysis of the ClinVar database and functional variants in literature could be reapplied for other genes related to other diseases, helping in early diagnosis, the prognosis of affected patients, and genetic counseling for affected families.

## Supplementary material

The following online material is available for this article:

Table S1 - All variants with not provided (NP) information in TSC1 and TSC2 for each category.

Table S2 - TSC1 and TSC2 variants with clinical significance in ClinVar divided by molecular consequence and variation type.

Table S3 - TSC1 and TSC2 variants with clinical significance and no molecular consequence submitted in ClinVar.

Table S4 - TSC1 and TSC2 variants with clinical significance and no variation type submitter in ClinVar.
