# Supplementary material for: An overview of actionable and potentially actionable TSC1 and TSC2 germline variants in an online Database
Source: Genet Mol Biol. 2024 Feb 19;46(3 Suppl 1):e20230132. doi: 10.1590/1678-4685-GMB-2023-0132 (PMC10876083; doi:10.1590/1678-4685-GMB-2023-0132)
Supplement: Table S1 - [file 1415-4757-GMB-46-03-s1-e20230132-s1.pdf]

Genetics and Molecular Biology
https://doi.org/10.1590/1678-4685-GMB-2023-0132

Supplementary Material to "An Overview of actionable and potentially actionable *TSC1* and *TSC2* germline variants in an online Database"

Table S1 - All variants with not provided (NP) information in *TSC1* and *TSC2* for each category.

	TSC1	***TSC2***
**Types of variants with NP clinical significance**	**Number of Variants**	**Number of Variants**
Synonymous	15 (4.81%)	15 (1.71%)
Microsatellite	2 (0.64%)	5 (0.57%)
Indel	8 (2.56%)	21 (2.39%)
Nonsense	46 (14.74%)	78 (8.89%)
Splice site	43 (13.78%)	223 (25.43%)
UTR	10 (3.21%)	7 (0.80%)
Single allele	0 (0%)	1 (0.11%)
CNV	1 (0.32%)	0 (0%)
Deletion	102 (32.69%)	238 (27.14%)
Duplication	49 (15.71%)	114 (13.00%)
Insertion	11 (3.53%)	30 (3.42%)
Missense	25 (8.01%)	146 (16.65%)
Others	0 (0%)	1 (0.11%)
Total	312 (100%)	877 (100%)
**Types of variants with NP molecular consequence**	**Number of Variants**	**Number of Variants**
Synonymous	650 (51.55%)	1,668 (47.17%)
Splice site	518 (41.08%)	1,520 (42.99%)
CNV	0 (0%)	67 (1.89%)
Duplication	22 (1.74%)	75 (2.12%)
Deletion	21 (1.67%)	149 (4.21%)
Single allele	6 (0.48%)	6 (0.17%)
Insertion	17 (1.35%)	11 (0.31%)
Indel	6 (0.48%)	12 (0.34%)
UTR 5'	1 (0.08%)	3 (0.08%)
Translocation	1 (0.08%)	0 (0%)

	TSC1	*TSC2*
Microsatellite	17 (1.35%)	24 (0.68%)
Others	1 (0.08%)	1 (0.03%)
Total	1,261	3,536 (100%)
**Types of variants with NP variation type**	**Number of Variants**	**Number of Variants**
Duplication	0 (0%)	15 (13.16%)
Insertion	0 (0%)	1 (0.88%)
CNV	0 (0%)	1 (0.88%)
Indel	0 (0%)	2 (1.75%)
Inversion	2 (0%)	1 (0.88%)
Microsatellite	62 (0%)	33 (28.95%)
Single allele	0 (0%)	1 (0.88%)
Splice site	0 (0%)	26 (22.81%)
Total	64	114 (100%)
