# Supplementary material for: An overview of actionable and potentially actionable TSC1 and TSC2 germline variants in an online Database
Source: Genet Mol Biol. 2024 Feb 19;46(3 Suppl 1):e20230132. doi: 10.1590/1678-4685-GMB-2023-0132 (PMC10876083; doi:10.1590/1678-4685-GMB-2023-0132)
Supplement: Table S2 - [file 1415-4757-GMB-46-03-s1-e20230132-s2.pdf]

Genetics and Molecular Biology
https://doi.org/10.1590/1678-4685-GMB-2023-0132

Supplementary Material to "An Overview of actionable and potentially actionable *TSC1* and *TSC2* germline variants in an online Database"

Table S2 - *TSC1* and *TSC2* variants with clinical significance in ClinVar divided by molecular consequence and variation type.

TSC1				
	**Clinical Significance**			
**Molecular consequence**	**Pathogenic/Likely pathogenic**	**Benign/Likely benign**	**Conflicting Submissions**	**VUS**
Frameshift	198 (39.68%)	0 (0%)	0 (0%)	12 (0.78%)
Missense	14 (2.81%)	70 (6.27%)	160 (69.57%)	1,111 (72.47%)
Nonsense	130 (26.05%)	0 (0%)	0 (0%)	2 (0.13%)
Splice site	68 (13.63%)	1 (0.09%)	1 (0.43%)	6 (0.39%)
UTR	0 (0%)	115 (10.30%)	3 (1.30%)	133 (8.68%)
Missense+UTR	4 (0.80%)	3 (0.27%)	12 (5.22%)	100 (6.52%)
Missense+Splice	0 (0%)	0 (0%)	0 (0%)	1 (0.07)
Frameshift+UTR	14 (2.81%)	0 (0%)	0 (0%)	0 (0%)
Nonsense+UTR	9 (1.80%)	0 (0%)	0 (0%)	0 (0%)
Not provided	62 (12.42%)	927 (83.06%)	54 (23.48%)	169 (11.02%)
Total	499 (100%)	1,116 (100%)	230 (100%)	1,534 (100%)
**Variation type**				
Deletion	162 (32.46%)	27 (2.42%)	2 (0.87%)	32 (2.09%)
Duplication	31 (6.21%)	0 (0%)	0 (0%)	3 (0.20%)
Single nucleotide	200 (40.08%)	1,053 (94.35%)	222 (96.52%)	1,437 (93.74%)
Insertion	13 (2.61%)	5 (0.45%)	1 (0.43%)	6 (0.39%)
Indel	15 (3.01%)	2 (0.18%)	2 (0.87%)	7 (0.46%)
Insertion+Duplication	58 (11.62%)	21 (1.88%)	2 (0.87%)	32 (2.09%)
Not Provided	20 (4.01%)	8 (0.72%)	1 (0.43%)	16 (1.04%)
Total	499 (100%)	1,116 (100%)	230 (100%)	1,534 (100%)

TSC2				
	**Clinical Significance**			
**Molecular consequence**	**Pathogenic/Likely pathogenic**	**Benign/Likely benign**	**Conflicting Submissions**	**VUS**
Frameshift	337 (32.81%)	2 (0.07%)	3 (0.45%)	15 (0.47%)
Missense	108 (10.52%)	165 (5.99%)	411 (61.90%)	2,547 (80.20%)
Nonsense	198 (19.28%)	2 (0.07%)	0 (0%)	11 (0.35%)
Splice site	183 (17.82%)	3 (0.11%)	6 (0.90%)	36 (1.13%)
UTR	0 (0%)	80 (2.90%)	8 (1.20%)	12 (0.38)
Missense+UTR	1 (0.10%)	5 (0.18%)	6 (0.90%)	115 (3.62%)
Missense+Splice	0 (0%)	0 (0%)	1 (0.15%)	4 (0.13%)
Frameshift+UTR	16 (1.56%)	0 (0%)	0 (0%)	0 (0%)
Nonsense+UTR	7 (0.68%)	0 (0%)	0 (0%)	0 (0%)
Not provided	177 (17.23%)	2,499 (90.67%)	229 (34.49%)	436 (13.73%)
Total	1027 (100%)	2,756 (100%)	664 (100%)	3,176 (100%)
**Variation type**				
Deletion	331 (32.23%)	58 (2.10%)	3 (0.45%)	76 (2.39%)
Duplication	27 (2.63%)	12 (0.44%)	0 (0%)	11 (0.35%)
Single nucleotide	455 (44.30%)	2,612 (94.78%)	646 (97.29%)	2,965 (93.36%)
Insertion	14 (1.36%)	3 (0.11%)	0 (0%)	4 (0.13%)
Indel	26 (2.53%)	9 (0.33%)	2 (0.30%)	32 (1.01%)
Insertion+Duplication	134 (13.05%)	38 (1.38%)	4 (0.60%)	69 (2.17%)
Not Provided	40 (3.89%)	24 (0.87%)	9 (1.36%)	19 (0.60%)
Total	1,027 (100%)	2,756 (100%)	664 (100%)	3,176 (100%)
