# Supplementary material for: An overview of actionable and potentially actionable TSC1 and TSC2 germline variants in an online Database
Source: Genet Mol Biol. 2024 Feb 19;46(3 Suppl 1):e20230132. doi: 10.1590/1678-4685-GMB-2023-0132 (PMC10876083; doi:10.1590/1678-4685-GMB-2023-0132)
Supplement: Table S3 - [file 1415-4757-GMB-46-03-s1-e20230132-s3.pdf]

Genetics and Molecular Biology
https://doi.org/10.1590/1678-4685-GMB-2023-0132

Supplementary Material to "An Overview of actionable and potentially actionable *TSC1* and *TSC2* germline variants in an online Database"

Table S3 - *TSC1* and *TSC2* variants with clinical significance and no molecular consequence submitted in ClinVar.

	TSC1	*TSC2*
	**Benign and Likely Benign**	
**Excel terms used for filtering**	**Number of Variants**	**Number of Variants**
Synonymous	585 (63.11%)	1,468 (58.74%)
Splice site	336 (36.25%)	1,012 (40.50%)
Copy number variation	0 (0%)	14 (0.56%)
Duplication	0 (0%)	0 (0%)
Deletion	0 (0%)	1 (0.04%)
Single allele	3 (0.32%)	0 (0%)
Insertion	1 (0.11%)	0 (0%)
Indel	1 (0.11%)	1 (0.04%)
UTR 5'	1 (0.11%)	0 (0%)
Translocation	0 (0%)	0 (0%)
Microsatellite	4 (0.43%)	3 (0.12%)
Promoter	4 (0.43%)	0 (0%)
Others	0 (0%)	0 (0%)
Total	927 (100%)	2,499 (100%)
	**Pathogenic and Likely Pathogenic**	
**Excel terms used for filtering**	**Number of Variants**	**Number of Variants**
Synonymous	0 (0%)	3 (1.69%)
Splice site	4 (6.45%)	24 (13.56%)
Copy number variation	31 (50%)	42 (23.73%)
Duplication	1 (1.61%)	9 (5.08%)
Deletion	20 (32.26%)	90 (50.85%)
Single allele	2 (3.23%)	2 (1.13%)
Insertion	3 (4.84%)	1 (0.56%)
Indel	0 (0%)	1 (0.56%)

UTR 5'	0 (0%)	1 (0.56%)
Translocation	0 (0%)	0 (0%)
Microsatellite	0 (0%)	4 (2.26%)
Promoter	0 (0%)	0 (0%)
Others	1 (1.61%)	0 (0%)
Total	62 (100%)	177 (100%)
	**Conflicting Submissions Variants**	
**Excel terms used for filtering**	**Number of Variants**	**Number of Variants**
Synonymous	26 (48.15%)	122 (53.28%)
Splice site	26 (48.15%)	99 (43.23%)
Copy number variation	0 (0%)	0 (0%)
Duplication	0 (0%)	2 (0.87%)
Deletion	1 (1.85%)	0 (0%)
Single allele	0 (0%)	0 (0%)
Insertion	0 (0%)	0 (0%)
Indel	0 (0%)	1 (0.44%)
UTR 5'	0 (0%)	0 (0%)
Translocation	0 (0%)	0 (0%)
Microsatellite	1 (1.85%)	5 (2.18%)
Promoter	0 (0%)	0 (0%)
Others	0 (0%)	0 (0%)
Total	54 (100%)	229 (100%)
	**Variants of Uncertain Significance**	
**Excel terms used for filtering**	**Number of Variants**	**Number of Variants**
Synonymous	0 (0%)	60 (13.76%)
Splice site	98 (57.99%)	257 (58.94%)
Copy number variation	4 (2.37%)	11 (2.52%)
Duplication	22 (13.02%)	55 (12.61%)
Deletion	6 (3.55%)	35 (8.03%)
Single allele	25 (14.79%)	1 (0.23%)
Insertion	0 (0%)	4 (0.92%)
Indel	0 (0%)	5 (1.15%)
UTR 5'	0 (0%)	0 (0%)
Translocation	0 (0%)	0 (0%)

Microsatellite	12 (7.10%)	8 (1.83%)
Promoter	2 (1.18%)	0 (0%)
Others	0 (0%)	0 (0%)
Total	169 (100%)	436 (100%)
