# Supplementary material for: An overview of actionable and potentially actionable TSC1 and TSC2 germline variants in an online Database
Source: Genet Mol Biol. 2024 Feb 19;46(3 Suppl 1):e20230132. doi: 10.1590/1678-4685-GMB-2023-0132 (PMC10876083; doi:10.1590/1678-4685-GMB-2023-0132)
Supplement: Table S4 - [file 1415-4757-GMB-46-03-s1-e20230132-s4.pdf]

Genetics and Molecular Biology
https://doi.org/10.1590/1678-4685-GMB-2023-0132

Supplementary Material to "An Overview of actionable and potentially actionable *TSC1* and *TSC2* germline variants in an online Database"

Table S4 - *TSC1* and *TSC2* variants with clinical significance and no variation type submitter in ClinVar.

	TSC1	***TSC2***
	**Benign and Likely Benign**	
**Excel terms used for filtering**	**Number of Variants**	**Number of Variants**
Deletion	0 (0%)	0 (0%)
Duplication	0 (0%)	0 (0%)
Insertion	0 (0%)	0 (0%)
SNV	0 (0%)	0 (0%)
Indel	0 (0%)	0 (0%)
CNV	0 (0%)	0 (0%)
Dup+Ins	0 (0%)	0 (0%)
Inversion	1 (12.50%)	0 (0%)
Single allele	0 (0%)	0 (0%)
Splice site	3 (37.50%)	14 (58.33%)
Translocation	0 (0%)	0 (0%)
Microsatellite	4 (50%)	10 (41.67%)
Total	8 (100%)	24 (100%)
	**Pathogenic and Likely Pathogenic**	
**Excel terms used for filtering**	**Number of Variants**	**Number of Variants**
Deletion	17 (85%)	22 (55%)
Duplication	2 (10%)	7 (17.50%)
Insertion	0 (0%)	1 (2.50%)
SNV	0 (0%)	0 (0%)
Indel	0 (0%)	2 (5%)
CNV	0 (0%)	1 (2.50%)
Dup+Ins	0 (0%)	0 (0%)
Inversion	0 (0%)	0 (0%)
Single allele	0 (0%)	0 (0%)

Splice site	0 (0%)	3 (7.50%)
Translocation	1 (5%)	0 (0%)
Microsatellite	0 (0%)	4 (10%)
Total	20 (100%)	40 (100%)

	Conflicting Submissions Variants	
**Excel terms used for filtering**	**Number of Variants**	**Number of Variants**
Deletion	0 (0%)	1 (11.11%)
Duplcation	0 (0%)	1 (11.11%)
Insertion	0 (0%)	0 (0%)
SNV	0 (0%)	2 (22.22%)
Indel	0 (0%)	0 (0%)
CNV	0 (0%)	0 (0%)
Dup+Ins	0 (0%)	0 (0%)
Inversion	0 (0%)	0 (0%)
Single allele	0 (0%)	0 (0%)
Splice site	0 (0%)	0 (0%)
Translocation	0 (0%)	0 (0%)
Microsatellite	1 (100%)	5 (55.56%)
Total	1 (100%)	9 (100%)

	Variants of Uncertain Significance	
**Excel terms used for filtering**	**Number of Variants**	**Number of Variants**
Deletion	0 (0%)	3 (15.79%)
Duplication	0 (0%)	0 (0%)
Insertion	0 (0%)	0 (0%)
SNV	0 (0%)	0 (0%)
Indel	0 (0%)	0 (0%)
CNV	0 (0%)	1 (5.26%)
Dup+Ins	0 (0%)	0 (0%)
Inversion	2 (12.50%)	1 (5.26%)
Single allele	1 (6.25%)	0 (0%)
Splice site	0 (0%)	5 (26.32%)
Translocation	0 (0%)	0 (0%)
Microsatellite	13 (81.25%)	9 (47.37%)
Total	16 (100%)	19 (100%)
